# Rosuvastatin Attenuates Pulmonary Damage in Rats with Cecal Ligation and Puncture-Induced Sepsis

**DOI:** 10.3390/jcm15114112

**Published:** 2026-05-26

**Authors:** Safiye İnşira Yıldız, Faruk Saydam, Atilla Topçu, Levent Tümkaya, Eda Yılmaz Kutlu, Hüseyin Avni Uydu

**Affiliations:** 1 Health Institutes of Türkiye (TÜSEB), Ankara 06800, Türkiye; s.insirah.yldz@gmail.com; 2 Department of Medical Biology, Faculty of Medicine, Recep Tayyip Erdogan University, Rize 53200, Türkiye; 3 Department of Pharmacology, Faculty of Medicine, Recep Tayyip Erdogan University, Rize 53200, Türkiye; atilla.topcu@erdogan.edu.tr; 4 Department of Histology and Embryology, Faculty of Medicine, Ondokuz Mayıs University, Samsun 55200, Türkiye; levent.tumkaya@omu.edu.tr; 5 Department of Medical Biochemistry, Faculty of Medicine, Recep Tayyip Erdogan University, Rize 53200, Türkiye; eda.yilmazkutlu@erdogan.edu.tr; 6 Department of Medical Biochemistry, Faculty of Medicine, Samsun University, Samsun 55080, Türkiye; huseyin.uydu@samsun.edu.tr

**Keywords:** sepsis, inflammation, rosuvastatin, lung, rat

## Abstract

**Background/Objectives:** Sepsis is a life-threatening syndrome arising from a dysregulated host response to infection, frequently leading to multiple organ dysfunction, with the lungs being among the most severely affected organs. Oxidative stress, inflammation, apoptosis, and DNA damage play key roles in the pathogenesis of sepsis-induced acute lung injury (ALI). Beyond its lipid-lowering effects, rosuvastatin possesses anti-inflammatory and antioxidant properties that may confer protective effects in sepsis. This study was designed to investigate the dose-dependent prophylactic efficacy of rosuvastatin in mitigating pulmonary damage in rats with cecal ligation and puncture (CLP)-induced sepsis. **Methods:** Sprague–Dawley rats were randomly divided into six groups: Sham, Sham + rosuvastatin (10 mg/kg), Sham + rosuvastatin (20 mg/kg), CLP, CLP + rosuvastatin (10 mg/kg), and CLP + rosuvastatin (20 mg/kg). Rosuvastatin was administered via oral gavage 4 h before the surgical procedures in the experimental groups. All animals were sacrificed 16 h following surgical procedures. Lung tissues were analyzed for biochemical markers, including malondialdehyde (MDA) and reduced glutathione (GSH), as well as histopathological changes and immunohistochemical expression of NF-κB/p65, caspase-3, and 8-OHdG. **Results:** CLP-induced sepsis significantly increased MDA levels while decreasing GSH levels, indicating enhanced oxidative stress. Rosuvastatin treatment significantly reversed these changes. Histopathological analysis revealed marked lung injury in the CLP group, including alveolar inflammation, interstitial inflammation, vascular congestion, and increased alveolar septal thickness, all of which were significantly reduced following rosuvastatin administration. Immunohistochemical findings demonstrated increased expression of NF-κB/p65, caspase-3, and 8-OHdG in the CLP group, whereas rosuvastatin significantly attenuated these expressions. No significant difference in prophylactic efficacy was observed between the 10 mg/kg and 20 mg/kg doses of rosuvastatin. **Conclusions:** Rosuvastatin demonstrated a protective effect against sepsis-induced pulmonary damage by reducing oxidative stress, inflammation, apoptosis, and DNA damage. These findings suggest that rosuvastatin may have prophylactic potential in sepsis; however, further support is needed from investigations of cellular pathways in different mechanistic directions.

## 1. Introduction

Sepsis is a potentially fatal condition that arises from an aberrant host response to infection, frequently resulting in multiple organ dysfunction and high global mortality rates [1]. Recent estimates suggest that sepsis is responsible for nearly 48.9 million cases and 11 million deaths globally each year [2]. Chronic inflammatory processes and organ failures constitute primary contributors to the elevated mortality rates associated with sepsis. The lungs are among the organs most commonly and severely involved in sepsis. A substantial number of patients develop acute lung injury (ALI), which may progress to acute respiratory distress syndrome (ARDS) if not effectively managed [3]. It has been reported that monocytes and macrophages contribute to the pathogenesis of sepsis by mounting an immune response against pathogens through the expression of several pro-inflammatory cytokines, including tumor necrosis factor-alpha (TNF-α), interleukin-6 (IL-6), interleukin-1 beta (IL-1β), and interleukin-10 (IL-10) [4]. Reactive oxygen species (ROS), including superoxide anions, hydrogen peroxide, peroxynitrite, hypochlorous acid, and hydroxyl radicals, promote the expression of these cytokines primarily through activation of the nuclear factor kappa B (NF-κB) signaling pathway. Moreover, activated pro-inflammatory cytokines upregulate the expression of ROS-generating enzymes, thereby promoting excessive ROS production [5]. Antioxidant defense systems counteract excessive ROS by enzymatically detoxifying reactive intermediates, principally through the coordinated actions of superoxide dismutase (SOD), glutathione reductase (GR), glutathione peroxidase (GSH-Px), and catalase (CAT). Dysregulated cytokine overproduction, in turn, disrupts cellular energy metabolism and compromises endothelial integrity, leading to increased vascular permeability and impaired tissue perfusion [6].

Apoptosis in lung tissue during sepsis develops because of structural and functional damage induced by an excessive inflammatory response affecting the alveolar epithelium and pulmonary microvascular endothelium. During sepsis, circulating mediators such as tumor TNF-α, IL-1β, IL-6, Fas ligand, and others activate both the extrinsic death receptor-mediated apoptotic pathway and the intrinsic mitochondrial stress-associated pathway; the convergence point of these pathways is the activation of caspase-3 [7,8]. Apoptosis of type I and type II pneumocytes disrupts alveolar fluid clearance and impairs gas exchange, whereas endothelial cell apoptosis promotes increased vascular permeability, interstitial and alveolar edema, and inflammatory cell infiltration [9].

During sepsis, activated neutrophils, macrophages, and injured pulmonary cells generate excessive amounts of ROS and reactive nitrogen species (RNS); these molecules induce oxidative damage to nuclear and mitochondrial DNA, including base oxidation, single- and double-strand breaks, and other oxidative modifications [10,11]. In this context, 8-hydroxy-2′-deoxyguanosine (8-OHdG) is one of the most used biomarkers and is widely recognized as a reliable indicator of oxidative DNA damage [12]. Pulmonary DNA damage, reflected by increased 8-OHdG levels and mitochondrial genomic injury, is closely associated with the induction of apoptosis; this process ultimately disrupts alveolar epithelial and endothelial integrity, promotes edema formation and impaired gas exchange, and may progress to ALI and ARDS [13].

Despite advances in supportive care, effective therapies specifically targeting sepsis-induced pulmonary injury remain limited, underscoring the need for novel interventions [14]. Experimental animal models are essential for studying sepsis pathophysiology and evaluating therapeutic agents. The cecal ligation and puncture (CLP) model is considered the standard in rodents, as it closely replicates the polymicrobial infection and systemic inflammation seen in humans. The CLP model reliably induces bacteremia, cytokine storm, and organ dysfunction, making it highly suitable for preclinical studies of sepsis and organ-protective strategies [15].

Beyond their lipid-lowering action, statins are recognized for a range of pleiotropic effects, including anti-inflammatory, antioxidant, immunomodulatory, and endothelial-protective properties. These biological actions suggest their potential as therapeutic agents in inflammatory pathologies such as sepsis [16,17]. Preclinical and clinical studies indicate that statins may improve outcomes in sepsis by modulating inflammatory pathways, reducing oxidative stress, and preserving organ function [18,19]. Rosuvastatin is a widely prescribed 3-hydroxy-3-methylglutaryl-CoA (HMG-CoA) reductase inhibitor, primarily used for the treatment of dyslipidemia and to reduce cardiovascular risk. Compared to other molecules in the same class, rosuvastatin has been reported to exhibit high efficacy in improving lipid profiles. Through its anti-inflammatory, antioxidant, and antithrombotic effects, it serves as an important agent for both primary and secondary prevention of cardiovascular disease [20]. These properties of rosuvastatin have been demonstrated to attenuate oxidative stress and systemic inflammation in various experimental animal models [21,22]. In lung tissue exposed to LPS-induced inflammation, rosuvastatin significantly decreased proinflammatory enzymes such as iNOS and COX-2 and reduced histopathological damage. Furthermore, the study demonstrated a marked decrease in the concentrations of key cytokines, including TNF-α, IL-6, and IL-1β, in both plasma and tissue samples [23]. However, the specific effects of rosuvastatin on sepsis-induced pulmonary injury, particularly in the CLP model, have not been fully characterized. This study aims to evaluate the protective effects of rosuvastatin on lung tissue in rats with sepsis induced by CLP, by analyzing key cellular parameters involved in oxidative stress, apoptosis, and inflammatory pathways.

## 2. Materials and Methods

### 2.1. Experimental Animals

Ethical approval for this study was obtained from the Local Ethics Committee for Animal Experiments of Recep Tayyip Erdoğan University (Approval Date: 16 July 2020; Decision No: 2020/32). The Scientific Research Projects Coordination Unit of Recep Tayyip Erdoğan University provided financial support for this research. Male Sprague–Dawley rats, 4–6 weeks of age and weighing 270–300 g, were used in this study. The animals were bred and housed at the Laboratory Animal Production and Research Center of Recep Tayyip Erdoğan University. All animals were maintained under standardized hygienic conditions in the experimental animal facility, with a controlled 12 h light/12 h dark cycle, a relative humidity of 55–60%, and an ambient temperature of 22 ± 2 °C. Throughout the study period, the animals were provided ad libitum access to tap water and standard pellet feed.

### 2.2. Treatment Groups

A total of 60 rats were randomly assigned to six groups, with 10 animals per group, as follows: Sham, Sham + Rosuvastatin (10 mg/kg), Sham + Rosuvastatin (20 mg/kg), CLP, CLP + Rosuvastatin (10 mg/kg), and CLP + Rosuvastatin (20 mg/kg). The group size was determined based on previous comparable experimental sepsis studies [4,24,25]. In the Sham groups, a laparotomy was conducted by carefully exteriorizing the cecum, performing minimal manipulation, and subsequently returning it to the abdominal cavity before surgical closure. Sepsis was induced in rats using the CLP method, and substance administrations were performed 4 h before the surgical procedures, as previously described in the literature [24]. The Sham + Rosuvastatin groups received oral gavage of rosuvastatin (Crestor, AstraZeneca İlaç. San. ve Tic. Ltd. Şti., Istanbul, Türkiye) at 10 or 20 mg/kg. By contrast, the sham control group was administered an equivalent volume of 0.9% NaCl. Rats in the CLP + Rosuvastatin groups were administered rosuvastatin at 10 or 20 mg/kg via oral gavage, while the CLP control group received 0.9% NaCl. All animals were monitored postoperatively under standard laboratory conditions until the experimental endpoint, which was set at 16 h following surgery administration. Sixteen hours after the surgical procedure, all animals were sacrificed under deep anesthesia induced by high-dose ketamine hydrochloride (Pfizer İlaçları Ltd. Şti., Istanbul, Türkiye) and xylazine hydrochloride (Rompun, Bayer, Hanover, NJ, USA). The lung tissues were subsequently bisected longitudinally. One portion was preserved at −80 °C for biochemical analyses, while the other was fixed in 10% neutral-buffered formalin (Sigma-Aldrich, Saint Louis, MO, USA) for histological evaluation. Predefined exclusion criteria included death before completion of the protocol, major procedure-related complications, insufficient sample volume, gross hemolysis, sample degradation, technical failure during biochemical analysis, or procedurally invalid values. No animals or samples met these criteria.

### 2.3. Cecal Ligation and Puncture (CLP) Model

All operative procedures were performed under sterile conditions. Anesthesia was induced by intraperitoneal administration of ketamine hydrochloride (50 mg/kg) and xylazine hydrochloride (10 mg/kg). After adequate anesthesia had been established, a 2.5 cm midline laparotomy was performed. The cecum was carefully exteriorized, and a ligation was performed just distal to the ileocecal valve using 4/0 silk sutures. Two punctures were then created in the distal cecum with a 22-gauge needle to allow the release of cecal contents into the peritoneal cavity. Following this, the cecum was returned to the abdominal cavity, and the incision was closed in two layers using sterile, absorbable 4/0 sutures. The surgical site was irrigated with 1% lidocaine (Onfarma, Samsun, Türkiye) to provide local analgesia.

### 2.4. Biochemical Analysis

#### 2.4.1. Tissue Homogenization

A homogenization solution consisting of 20 mM sodium phosphate and 140 mM potassium chloride (KCl) at pH 7.4 was initially prepared. Subsequently, 1 mL of homogenization solution was added to 0.1 g of the tissue sample, and the mixture was then homogenized. The homogenates were subjected to centrifugation at 800 *g* for 10 min at 4 °C. The supernatants obtained were used to assess thiobarbituric acid-reactive substances (TBARS) and –SH levels.

#### 2.4.2. TBARS Analysis

TBARS, a significant marker of tissue damage, serves as a critical indicator of oxidant stress that activates reactive oxygen species (ROS) through the oxidative stress pathway. This procedure was conducted according to the method outlined by Ohkawa et al. [26]. Briefly, 200 μL of tissue supernatant was mixed with 50 μL of 8.1% sodium dodecyl sulfate, 375 μL of 20% acetic acid (*v*/*v*; pH 3.5), and 375 μL of 0.8% thiobarbituric acid (TBA). The reaction mixture was vortexed, heated in a boiling water bath for 1 h, cooled in ice water for 5 min, and then centrifuged at 750× *g* for 10 min. The absorbance of the resulting pink chromogen was measured spectrophotometrically at 532 nm, and the findings were expressed as nmol/mg tissue.

#### 2.4.3. Total Thiol Group Analysis

Total –SH groups were quantified using Ellman’s reagent (Merck KGaA, Darmstadt, Germany) [27]. Briefly, 100 μL of 3 M Na_2_HPO_4_ and 25 μL of DTNB (5,5-dithiobis-(2-nitrobenzoic acid)) were added to 25 μL of supernatant. The DTNB solution contained 4 mg of DTNB dissolved in 10 mL of 1% sodium citrate solution. Following gentle mixing, the resulting yellow coloration was measured spectrophotometrically at 412 nm. Quantification was performed using a standard curve generated from reduced glutathione concentrations ranging from 1000 μM to 62.5 μM, and the results were expressed as nmol/mg tissue.

### 2.5. Histopathological Analysis

Lung samples were fixed in 10% neutral-buffered formalin (Sigma-Aldrich, Saint Louis, MO, USA) for 24 h. Following fixation, the tissues were dehydrated through a graded ethanol (Merck GmbH, Darmstadt, Germany) series according to standard histological protocols. Subsequently, the specimens were cleared in two changes of xylene (Merck GmbH, Darmstadt, Germany) and embedded in paraffin blocks (Isolab GmbH, Eschau, Germany) using a tissue embedding system (Leica EG1150 H, Leica Biosystems Nussloch GmbH, Nussloch, Germany). The sections were subsequently stained with Hematoxylin–Eosin (H&E, Harris hematoxylin Eosin G Merck GmbH, Darmstadt, Germany) using an automated tissue stainer (Leica ST5020, Leica Microsystems Nussloch GmbH, Nussloch, Germany). Following staining, the samples were examined under a light microscope (Olympus BX51, Olympus Corp., Tokyo, Japan), and images were captured with a digital camera mounted on the microscope.

**Figure 1:**
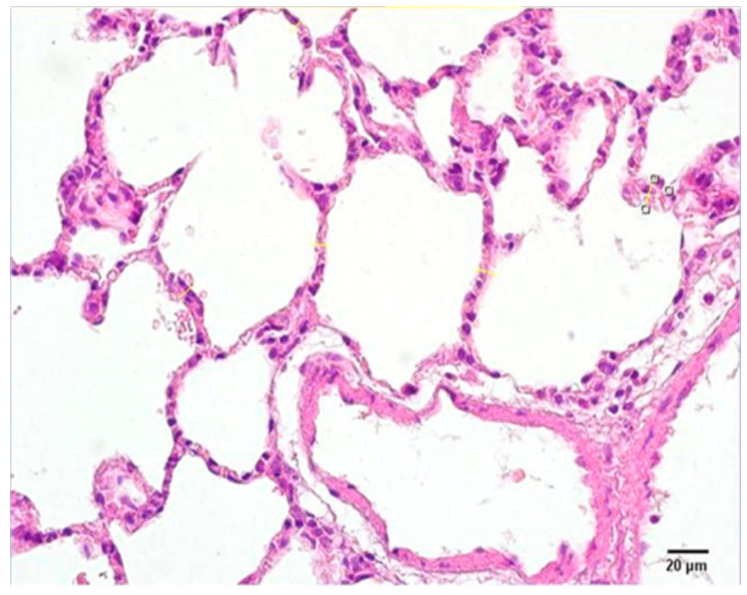
The Olympus DP 2.0 (Olympus Corp., Japan) software program, by which alveolar septal wall thickness was measured in tissue section images using an arbitrary probe.

Histopathological evaluation of H&E-stained sections was performed by one histopathologist, blinded to the experimental groups, who examined and scored thirty-five distinct microscopic fields for each animal. Lung tissue samples were evaluated for alveolar inflammation, interstitial inflammation, vascular congestion, and alveolar septal thickening. Alveolar septal wall thickness in the tissue sections was measured using the arbitrary probe tool of the Olympus DP 2.0 software (Figure 1, Olympus Corp., Japan). The lung damage score (LDS) was calculated according to the system described by Matute-Bello et al. [28]. In this scoring method, the extent of tissue damage was assigned a score of 0, 1, 2, or 3 based on the percentage of affected tissue. Alveolar septal thickening was scored based on the ratio of each treatment group relative to the sham group (Table 1).

### 2.6. Immunohistochemical (IHC) Analysis

Primary antibodies were used to evaluate the inflammatory response (anti-NF-κB/p65, rabbit polyclonal, Abcam, ab16502, Cambridge, UK), to identify apoptotic pneumocytes (anti-caspase-3, rabbit polyclonal, Abcam, ab44976, UK), and to detect oxidative DNA damage (anti-8-OHdG [8-hydroxy-2-deoxyguanosine], rabbit polyclonal, Abcam, ab16502, UK). Detection was performed with an appropriate horseradish peroxidase-conjugated secondary antibody (Goat Anti-Rabbit IgG H&L (HRP), Abcam, ab205718, UK). Lung tissue paraffin sections, 2–3 μm thick, were mounted on positively charged slides (Patolab Biomedikal, Istanbul, Türkiye). After dehydration, the sections were exposed to hydrogen peroxide to suppress endogenous peroxidase activity in accordance with the protocols specified by the primary antibody kit manufacturers. Following deparaffinization and antigen retrieval performed using the Leica IHC/ISH system (Leica Biosystems, Germany), the tissue sections were incubated with the respective primary and secondary antibodies for 60 min each, in accordance with the manufacturer’s standardized protocol. Immunoreactivity was visualized using diaminobenzidine tetrahydrochloride (Leica LTraview DAB, Leica Biosystems, Wetzlar, Germany), and the sections were counterstained with Harris hematoxylin (Merck, Darmstadt, Germany).

Immunopositive cells in the IHC-stained sections were evaluated using a semi-quantitative scoring system (Table 2). One histopathologist, blinded to the experimental groups, independently assessed thirty-five randomly selected regions per section at 40× magnification.

### 2.7. Statistical Analysis

The histological and biochemical findings obtained from the lung tissue samples were statistically evaluated using IBM SPSS Statistics version 23 (SPSS Inc., Chicago, IL, USA). The Shapiro–Wilk test and Levene’s test were used to assess data normality and homogeneity of variances. Data showing normal distribution and homogeneity of variance were presented as mean ± standard deviation (SD) and analyzed by one-way ANOVA with Tukey’s HSD post hoc test. Semi-quantitative and non-parametric data were presented as median (interquartile range) and analyzed using the Kruskal–Wallis test followed by Dunn’s post hoc multiple-comparison test. A *p*-value of <0.05 was considered statistically significant.

## 3. Results

### 3.1. Malondialdehyde (MDA) Levels

When MDA levels were analyzed, they were found to be significantly higher in the CLP group compared to the Sham group (*p* < 0.05). MDA levels were significantly reduced in both the CLP + 10 mg/kg rosuvastatin and CLP + 20 mg/kg rosuvastatin groups relative to the CLP group (*p* < 0.05). No statistically significant difference was observed between the two rosuvastatin doses (10 mg/kg and 20 mg/kg). Detailed MDA data are presented in Table 3.

### 3.2. Reduced Glutathione (GSH) Levels

The analysis of GSH levels revealed a significant decrease in the CLP group compared to the Sham group (*p* < 0.05). Rosuvastatin treatment significantly increased GSH levels in the CLP groups (*p* < 0.05). No significant difference was observed between the two doses of rosuvastatin (10 mg/kg and 20 mg/kg). The GSH data are presented in Table 4.

### 3.3. Histopathological Findings

**Figure 2:**
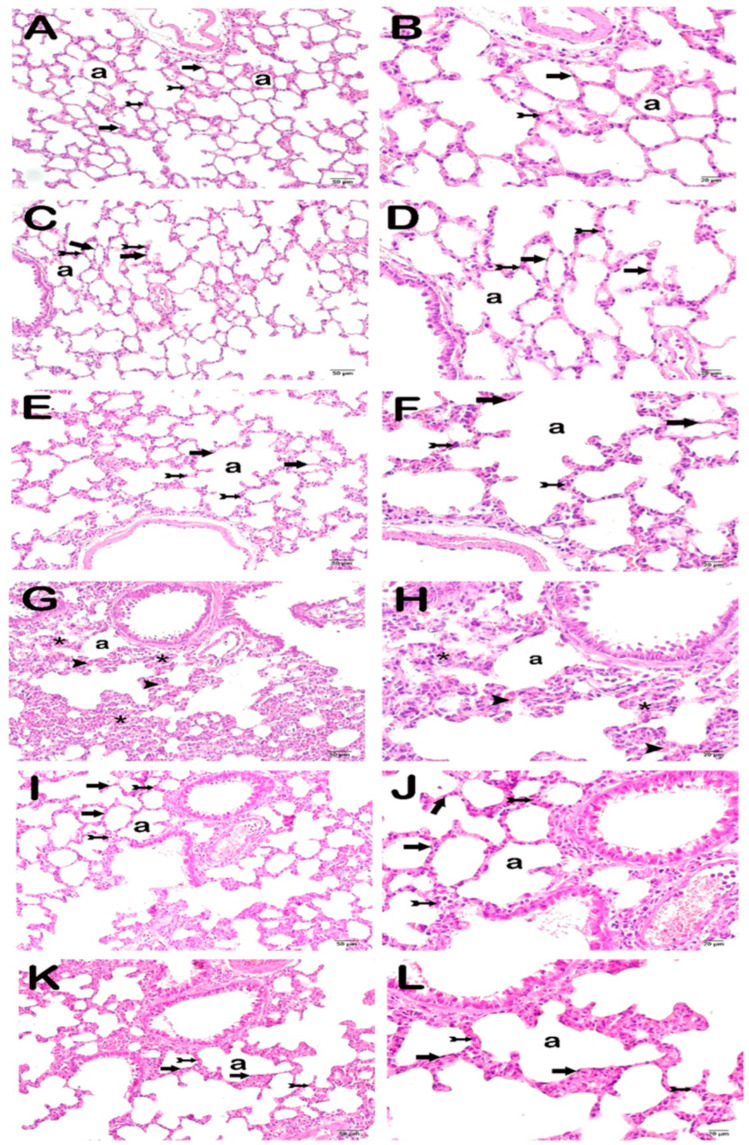
Representative light microscopic image of lung tissue sections stained with Harris hematoxylin and eosin G. Alveolus (a), type I pneumocyte (arrow), and type II pneumocyte (arrowhead). (**A**) (×20)–(**B**) (×40): In sections from the healthy control group, alveolar septal walls composed of normal type I pneumocytes (arrow) and type II pneumocytes were observed (LDS: 0 [0–0.5]). (**C**) (×20)–(**D**) (×40): In sections from the CR10 mg treatment group, alveolar septal wall structures with typical morphology were observed (LDS: 0 [0–1]). (**E**) (×20)–(**F**) (×40): In sections from the CR20 mg treatment group, alveolar (a) structures composed of morphologically normal type I pneumocytes (arrow) and type II pneumocytes (arrowhead) were observed (LDS: 1 [0–1]). (**G**) (×20)–(**H**) (×40): In sections from the CLP group, thickening of the alveolar septal walls (asterisk) was observed. In addition, widespread vascular congestion (arrowhead) was detected in the interstitial areas (LDS: 5 [4,5,6,7]). (**I**) (×20)–(**J**) (×40): In sections from the CLP + CR10 mg treatment group, a reduction in alveolar septal wall thickness and decreased vascular congestion in the interstitial areas were observed. In addition, alveolar (a) structures composed of morphologically typical type I pneumocytes (arrow) and type II pneumocytes (arrowhead) were identified (LDS: 0.5 [0–2]). (**K**) (×20)–(**L**) (×40): In sections from the CLP + CR20 mg treatment group, reduced alveolar septal wall thickness was observed, together with alveolar (a) structures composed of morphologically typical type I pneumocytes (arrow) and type II pneumocytes (arrowhead) (LDS: 1 [0–1]).

Representative microscopic images of lung tissue samples evaluated for alveolar inflammation, interstitial inflammation, vascular congestion, and alveolar septal thickness are presented in Figure 2. The findings were evaluated according to the LDS system proposed by Matute-Bello et al. [28]. The scores of the assessed lung tissue parameters were significantly higher in the CLP group compared to the other groups. Administration of rosuvastatin to septic rats resulted in a significant reduction in all scores. However, no significant dose-dependent difference was observed between the groups receiving 10 mg and 20 mg of rosuvastatin (Table 5).

Table 6 presents the alveolar septal wall thickness values of rat lung tissue as the arithmetic mean ± standard deviation (mean ± SD). The fold changes relative to the sham group were also determined. In the CLP group, septal wall thickness exhibited an almost threefold increase, whereas administration of rosuvastatin resulted in an average reduction of approximately 1.5-fold. Nevertheless, no statistically significant differences were observed among the various rosuvastatin dosage groups.

### 3.4. Immunohistochemical Results

The immunohistochemical positivity scores for the antibodies used are presented in Table 7. In the CLP group, the scores of all antibodies were significantly higher compared to the other groups. Administration of rosuvastatin in septic animal models resulted in a significant reduction in these scores. The positivity scores of NF-κB/p65, caspase-3, and 8-OHdG antibodies did not show an important difference between the 10 mg and 20 mg rosuvastatin groups.

**Figure 3:**
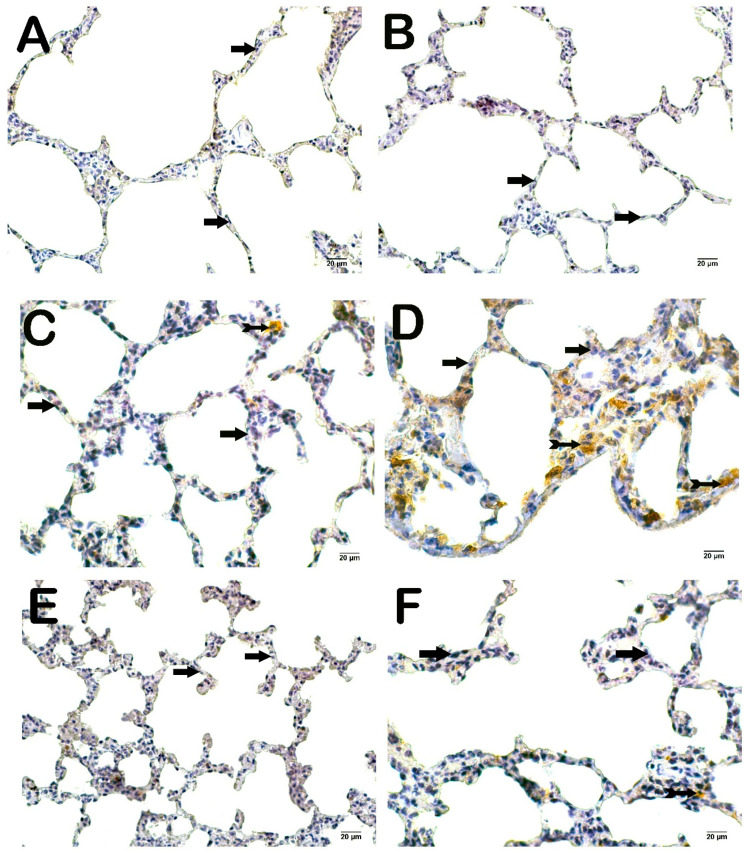
Light microscopic image of lung tissue sections incubated with caspase-3. Positive cells are marked with arrowheads, whereas normal cells are indicated by straight arrows. (**A**): Sham, (**B**): Sham + 10 mg/kg Rosuvastatin, (**C**): Sham + 20 mg/kg Rosuvastatin, (**D**): CLP, (**E**): CLP + 10 mg/kg Rosuvastatin, (**F**): CLP + 20 mg/kg Rosuvastatin.

Representative photomicrographs of lung sections immunolabeled for caspase-3 are shown in Figure 3. In sections from the CLP group, numerous pneumocytes within the alveolar septa exhibited intense caspase-3 immunoreactivity, with prominent cytoplasmic and occasional perinuclear localization consistent with apoptotic morphology (arrowheads). In contrast, lungs from rosuvastatin-treated septic rats contained markedly fewer caspase-3-positive cells, and staining intensity was substantially attenuated. ^a^ *p* < 0.001; Compared with the sham group. ^b^ *p* < 0.001; Compared with the CLP group. Kruskal–Wallis/Dunn test.

**Figure 4:**
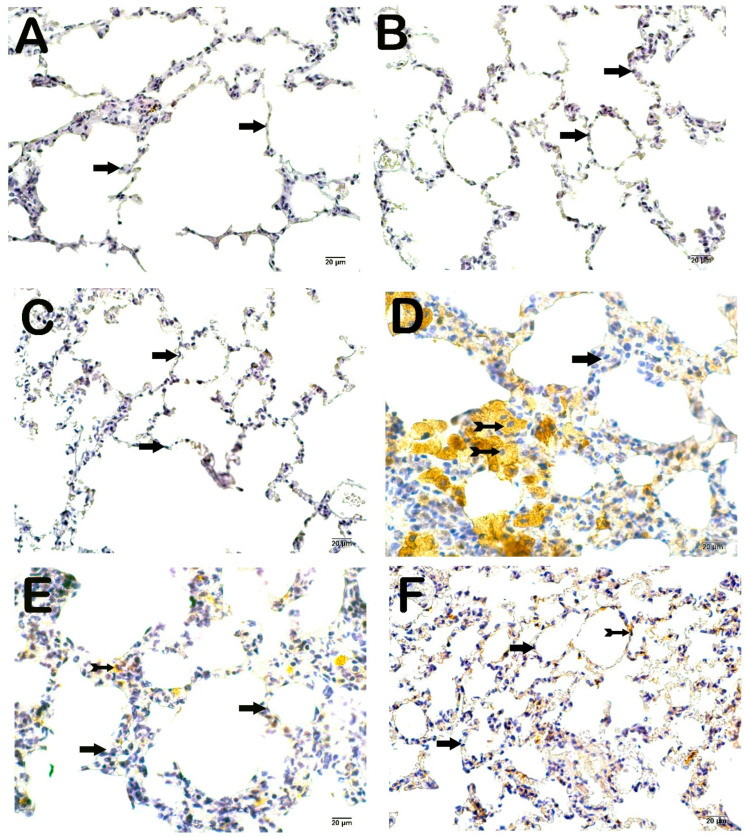
Light microscopic image of lung tissue sections incubated with NF-kβ/p65. Positive cells are marked with arrowheads, whereas normal cells are indicated by straight arrows. (**A**): Sham, (**B**): Sham + 10 mg/kg Rosuvastatin, (**C**): Sham + 20 mg/kg Rosuvastatin, (**D**): CLP, (**E**): CLP + 10 mg/kg Rosuvastatin, (**F**): CLP + 20 mg/kg Rosuvastatin.

Representative images of lung sections immunostained for NF-κB/p65 are presented in Figure 4. Sections from the CLP group showed abundant NF-κB/p65-immunopositive pneumocytes (arrowheads), whereas rosuvastatin-treated septic rats displayed a reduced number of NF-κB/p65-positive pneumocytes compared with the CLP group.

**Figure 5:**
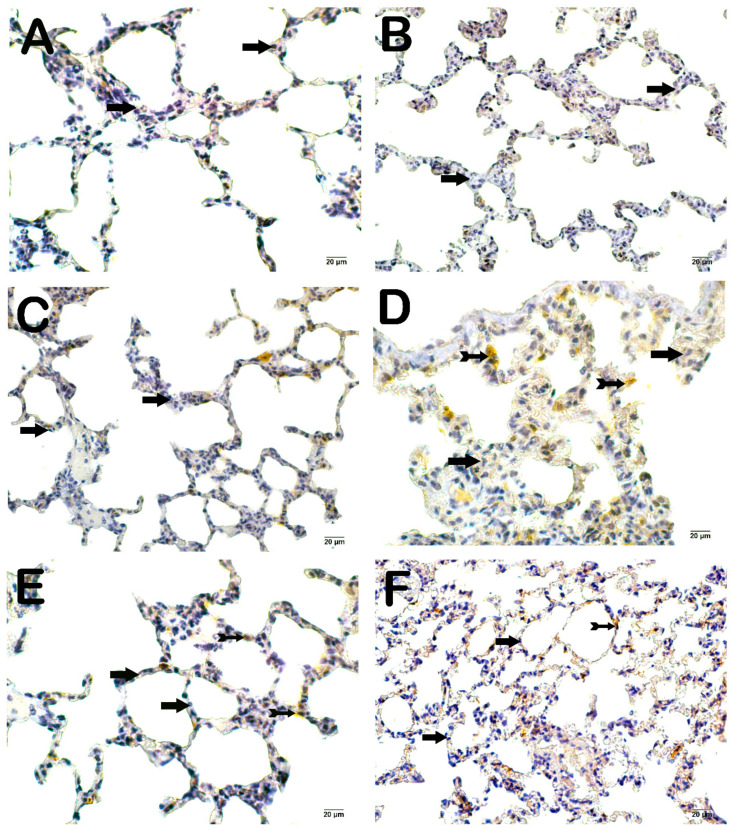
Light microscopic image of lung tissue sections incubated with 8-OHdG. Positive cells are marked with arrowheads, whereas normal cells are indicated by straight arrows. (**A**): Sham, (**B**): Sham + 10 mg/kg Rosuvastatin, (**C**): Sham + 20 mg/kg Rosuvastatin, (**D**): CLP, (**E**): CLP + 10 mg/kg Rosuvastatin, (**F**): CLP + 20 mg/kg Rosuvastatin.

Figure 5 shows representative images of lung tissue sections probed with anti-8-OHdG antibodies. The CLP group displayed the highest 8-OHdG signal, with expression significantly elevated relative to all other groups. In the rosuvastatin-treated groups, the number of 8-OHdG-positive pneumocytes was markedly reduced, and no significant differences were observed between the administered doses.

## 4. Discussion

Despite numerous proposed mechanisms, the pathophysiology of sepsis remains incompletely elucidated. Nevertheless, it is well established that a dysregulated host immune response to infection drives tissue injury. Immune cells recruited to sites of inflammation augment the synthesis and levels of pro-inflammatory cytokines, thereby precipitating cellular damage within organs [29]. Pro-inflammatory cytokines stimulate the production of reactive oxygen species (ROS), thereby inducing oxidative stress and consequent tissue injury. In sepsis, the lungs are a primary target of this injury and one of the most frequently affected organs. Malondialdehyde (MDA), a lipid peroxidation product and a key biomarker of tissue-level oxidative stress, has been reported to increase in the lungs following induction of sepsis [30]. Reduced glutathione (GSH) is the cell’s foremost non-enzymatic antioxidant. Serving primarily as an electron donor for glutathione peroxidase (GPx), it detoxifies peroxides and helps neutralize superoxide and hydroxyl radicals [31].

The documented antioxidant, anti-inflammatory, and immunomodulatory properties of statins have motivated their evaluation as adjunctive or alternative therapeutic strategies for chronic diseases. Rosuvastatin exhibits high affinity for the active site of HMG-CoA reductase and demonstrates superior in vitro potency in inhibiting enzymatic activity and cholesterol biosynthesis relative to other statins [21].

In rat models of sepsis induced by CLP, it has been reported that MDA levels in lung tissue are significantly increased, whereas GSH levels are decreased. Various compounds have been shown to attenuate sepsis-induced pulmonary injury by decreasing MDA levels and increasing GSH levels [4,32,33]. However, the potential effect of rosuvastatin on these oxidative stress parameters in lung tissue has not been clearly demonstrated in animal models of sepsis. In the present study, the elevated MDA levels and decreased GSH levels observed in the sepsis group were significantly reversed following rosuvastatin administration. These findings suggest that rosuvastatin exerts a protective effect against sepsis-induced oxidative damage in lung tissue.

In a study evaluating rosuvastatin’s antioxidant effects, rats were exposed to the toxicant fipronil to induce hepatic and renal oxidative injury. Abdel-Daim et al. administered rosuvastatin (10 mg/kg, oral, 15 days) and measured oxidative and antioxidant markers. Rosuvastatin significantly lowered fipronil-elevated MDA and restored depleted GSH in both organs. Fipronil also enhanced caspase-3 expression in hepatic and renal tissues; co-administration of oral rosuvastatin with vitamin E attenuated this apoptosis-associated upregulation [34]. In a separate study conducted in myocardial tissue, azithromycin administration elevated malondialdehyde (MDA) levels, which were reduced by maintenance-dose rosuvastatin (2 mg/kg), while glutathione (GSH) levels increased. Concurrently, the azithromycin-induced upregulation of caspase-3 expression was attenuated by rosuvastatin [35]. Consistent with these reports, rosuvastatin in our study conferred protection to lung tissue under heightened oxidative stress by reducing MDA levels and significantly increasing GSH. Thus, we confirm rosuvastatin’s antioxidant effect in sepsis-induced lung injury, extending prior evidence to pulmonary tissue.

In sepsis, NF-κB/p65 is central to TLR-mediated signaling: nuclear translocation of p65 triggers transcription of TNF-α, IL-1β, IL-6, and iNOS/COX-2. This program amplifies neutrophil recruitment, oxidative stress, and epithelial apoptosis, culminating in diffuse alveolar injury. Apoptosis of alveolar epithelial and capillary endothelial cells leads to barrier dysfunction, proteinaceous edema, and impaired gas exchange. Caspase-3, a principal executioner caspase, occupies a central position in the pathophysiology of sepsis. By integrating inputs from the intrinsic (caspase-9-dependent) and extrinsic (caspase-8-dependent) pathways, it orchestrates and carries out the terminal proteolytic events that execute the apoptotic program [36,37].

As one of the earliest organs affected during sepsis, the lungs undergo disruption of tissue integrity because of marked neutrophil infiltration within the pulmonary epithelium, leading to alveolar inflammation, interstitial inflammation, and vascular congestion. In addition, alveolar septal thickness is one of the histopathological features that is markedly altered and commonly evaluated in sepsis [28]. It has been reported that, in the CLP-induced sepsis group in which these histopathological features were analyzed, both the lung injury score (LDS) and alveolar septal thickness were significantly increased. [24,25]. In our histopathological analysis based on the scoring system of Matute-Bello et al., the lung injury score (LDS) was 0 (0–0.5) in the Sham group and 5 (4–7) in the CLP group. Following administration of rosuvastatin at 10 mg, this value decreased significantly to 0.5 (0–2), whereas treatment with 20 mg rosuvastatin reduced it to 1 (0–1). When the alveolar septal thickness ratio in the Sham group was accepted as 1, this ratio was determined to be 2.94 in the CLP group, 1.50 in the group treated with 10 mg rosuvastatin, and 1.60 in the group treated with 20 mg rosuvastatin. Our findings indicate that rosuvastatin exerts a protective effect against sepsis-induced acute lung injury (ALI); however, this effect appears to be dose-independent. Further studies are needed to validate and support these findings.

In a recently published study, increased expression of TNF-α and NF-κB in lung tissues of a rat sepsis model was reported [4]. Consistent findings have also been documented in earlier studies evaluating various compounds [32,38]. In a murine model of sepsis, increased TNF-α expression in cortical tissues has been reported, and rosuvastatin was shown to significantly attenuate this elevation [2]. Similarly, in the peritoneal lavage supernatants of the same model, elevated TNF-α levels were significantly reduced following rosuvastatin administration [15]. Mărginean et al. compared the anti-inflammatory effects of rosuvastatin and simvastatin in a rat model of CLP. The statins were administered 18 and 3 h before surgery, and serum levels of procalcitonin and cytokines (IL-1β, IL-6, and TNF-α) were measured. Both rosuvastatin and simvastatin demonstrated significant anti-inflammatory activity in this sepsis model [39]. In another study, lung tissue samples from the CLP-induced sepsis group exhibited markedly elevated NF-κB/p65 expression, which was significantly reduced following fosfomycin administration [24]. In our study, NF-κB/p65 levels were elevated in the sepsis group, whereas this increase was significantly attenuated in the rosuvastatin-treated group. These findings suggest that rosuvastatin may influence inflammation-related signaling through NF-κB/p65, although direct effects on downstream cytokines were not assessed.

8-OHdG is elevated in sepsis at both systemic (serum/urine) and pulmonary tissue levels, parallels indices of oxidative stress and lipid peroxidation (e.g., malondialdehyde, MDA), and correlates with disease severity and mortality. These findings support 8-OHdG as a reliable biomarker of oxidative DNA damage in sepsis and a useful pharmacodynamic readout for monitoring the impact of antioxidant or anti-inflammatory interventions [40]. In a study using a CLP-induced sepsis/ALI model in rats, 8-OHdG levels were evaluated in lung tissue. The authors reported that 8-OHdG levels were elevated in the CLP group, whereas resveratrol treatment attenuated this elevation by 41%. Another study employing the same model, in which 8-OHdG immunopositivity was assessed, likewise reported increased 8-OHdG levels in the sepsis group and demonstrated that costunolide treatment reduced this marker [41]. However, the effect of rosuvastatin on oxidative DNA damage in lung tissue has not previously been demonstrated. In our experimental groups, 8-OHdG levels were evaluated by immunohistochemical analysis. The increased positivity scores observed in the lung tissues of the sepsis group showed a statistically significant reduction following administration of rosuvastatin at doses of 10 mg and 20 mg. Nevertheless, no significant difference was detected between the two treatment doses. Our findings regarding 8-OHdG levels indicate that rosuvastatin also exerts a protective effect on the cellular pathways involved in oxidative DNA damage.

Rosuvastatin has been shown to inhibit apoptotic pathways in multiple tissues, including the liver, kidney, and myocardium [34,35]. Extending this evidence to the lung, we demonstrate an anti-apoptotic effect in the clinically relevant setting of sepsis, as evidenced by reduced caspase-3 expression. Caspase-3 levels, which were elevated in the CLP group, declined markedly after rosuvastatin treatment, without a discernible dose-dependent difference.

The absence of a significant difference between the 10 mg/kg and 20 mg/kg rosuvastatin doses with respect to the analyzed parameters in our study may be related to the potential saturation effects of the drug, the timing of administration, and pharmacokinetic limitations associated with oral delivery. Previous experimental studies with rosuvastatin have reported that the most preferred oral dose range in rat models is 10–20 mg/kg [42,43]. In addition to the first-pass effect associated with oral administration, this finding may also be related to the possibility that, as with many drugs, the oral bioavailability of rosuvastatin and its intracellular uptake are influenced by transporter-mediated mechanisms, such that the effective tissue exposure may not increase linearly despite dose escalation. Accordingly, a plateau response in pharmacodynamic efficacy beyond certain dose levels is likely. Similarly, in a study conducted by Saleh et al., a 10 mg/kg dose of rosuvastatin was reported to significantly suppress apoptotic pathways; however, the expected marked dose-dependent effect at higher doses could not be demonstrated [44]. In this context, further experimental studies evaluating lower (5 mg/kg) or higher doses may provide a more detailed understanding of the dose–response relationship of rosuvastatin, and our study may serve as a pilot investigation supporting such future research.

Our study had several limitations. First, sepsis is a systemic condition that affects not only the lungs but also the circulatory system (e.g., serum TNF-α, IL-6, MDA, GSH) and other organs, including the liver, kidneys, and spleen. Therefore, analysis of these organs would be necessary to comprehensively evaluate the overall effects of rosuvastatin. In addition, oxidative stress, inflammation, apoptosis, and oxidative DNA damage pathways were assessed using a limited number of biomarkers in the present study. Evaluation of additional key markers involved in these pathways would be required to further validate our findings. Moreover, tissue integrity was examined at the microscopic level; however, assessment of intercellular junction molecules at the molecular level could have provided more direct evidence regarding the effects of rosuvastatin. Another methodological limitation of our study is that the histological evaluations were performed by only one blinded histopathologist. Rosuvastatin was administered exclusively as a prophylactic treatment, 4 h before sepsis induction. Additional experimental designs incorporating post-sepsis treatment regimens would enhance the clinical relevance and translational value of our findings.

## 5. Conclusions

Although studies investigating the effects of rosuvastatin in experimental sepsis models remain limited, the available evidence suggests that this agent has anti-inflammatory potential. In this regard, our findings indicating the biochemical and histological protective effects of rosuvastatin in a CLP-induced sepsis model in rats are noteworthy. Nevertheless, in addition to further studies targeting a broader spectrum of cellular signaling pathways, therapeutic rather than prophylactic models are also required to fully elucidate the protective potential and clinical applicability of rosuvastatin in sepsis. Additionally, when the two different doses of rosuvastatin (10 mg/kg and 20 mg/kg) were compared across all analyzed parameters, no significant difference was observed between the dose groups. This finding indicates that rosuvastatin did not exhibit a dose-dependent effect under the conditions of the present study. Given the therapeutically challenging nature of sepsis, characterized by complex pathophysiology and high mortality, the demonstration of a prophylactic effect of a widely used drug such as rosuvastatin represents an important finding.

## Figures and Tables

**Table 1:** Lung Damage Score (LDS) as defined by Matute-Bello et al.

Findings	Score			
	0	1	2	3
Alveolar Inflammation	≤5%	6–25%	26–50%	≥50%
Interstitial Inflammation	≤5%	6–25%	26–50%	≥50%
Vascular Congestion	≤5%	6–25%	26–50%	≥50%
Alveolar Septal Thickening (Treatment Group/Sham Group Ratio)	≤×1	<×2	≥×2	≥×4

**Table 2:** Immunohistochemical staining positivity scores.

Findings	Score	Percentage%
None	0	<5%
Mild	1	5–25%
Moderate	2	26–50%
Severe	3	50%>

**Table 3:** Mean MDA levels of the experimental groups and the results of the statistical analysis.

Group	MDA (nmol/mg)
Sham	54.68 ± 1.6
CLP	92.56 ± 14.7 ^a^
CLP + 10 mg/kg Rosuvastatin	51.08 ± 6.5 ^b^
CLP + 20 mg/kg Rosuvastatin	57.40 ± 10.6 ^b^
Sham + 10 mg/kg Rosuvastatin	46.53 ± 3.2
Sham + 20 mg/kg Rosuvastatin	51.50 ± 4.7

Data are expressed as mean ± standard deviation. Statistical analysis was performed using one-way ANOVA followed by Tukey’s HSD test (^a^ *p* < 0.05 vs. Sham group; ^b^ *p* < 0.05 vs. CLP group).

**Table 4:** Mean GSH levels of the experimental groups and the results of the statistical analysis.

Group	GSH (nmol/mg)
Sham	19.05 ± 2.8
CLP	12.75 ± 1.7 ^a^
CLP + 10 mg/kg Rosuvastatin	19.53 ± 3.4 ^b^
CLP + 20 mg/kg Rosuvastatin	19.04 ± 3.8 ^b^
Sham + 10 mg/kg Rosuvastatin	18.72 ± 3.6
Sham + 20 mg/kg Rosuvastatin	17.35 ± 3.5

Data are expressed as mean ± standard deviation. Statistical analysis was performed using one-way ANOVA followed by Tukey’s HSD test (^a^ *p* < 0.05 vs. Sham group; ^b^ *p* < 0.05 vs. CLP group).

**Table 5:** Lung damage scores (LDS, Matute-Bello et al.) of the experimental groups (median with 25% and 75% interquartile range).

Group	Alveolar İnflammation	Interstitial Inflammation	Vascular Congestion	LDS
Sham	0 (0–0)	0 (0–0)	0 (0–0)	0 (0–0.5)
Sham + 10 mg/kg Rosuvastatin	0 (0–0)	0 (0–0) ^b^	0 (0–0) ^b^	0 (0–1) ^b^
Sham + 20 mg/kg Rosuvastatin	0 (0–0)	0 (0–1) ^c^	0 (0–1) ^b^	1 (0–1) ^b^
CLP	1 (0–1) ^a^	1 (1–2) ^a^	2 (1–2) ^a^	5 (4–7) ^a^
CLP + 10 mg/kg Rosuvastatin	0 (0–1)	0 (0–1) ^d^	0 (0–1) ^b^	0.5 (0–2) ^b^
CLP + 20 mg/kg Rosuvastatin	0 (0–0)	0 (0–1) ^e^	0 (0–0) ^b^	1 (0–1) ^b^

^a^ *p* < 0.001; Compared with the sham group. ^b^ *p* < 0.001; Compared with the CLP group. ^c^ *p* = 0.004; Compared with the CLP group. ^d^ *p* = 0.002; Compared with the CLP group. ^e^ *p* = 0.001; Compared with the CLP group. Kruskal–Wallis/Dunn test.

**Table 6:** Alveolar septum thickness.

Group	Alveolar Septal Wall Thickness (μm)	Alveolar Septum Thickness (Treatment Group/Control Group)	Alveolar Septum Thickness Score (Matute-Bello et al. [28])
Sham	8.06 ± 1.68	1	0 (<×2)
Sham + 10 mg/kg Rosuvastatin	9.91 ± ${2.15\;}^{b}$	1.22	0 (<×2)
Sham + 20 mg/kg Rosuvastatin	9.71 ± ${1.81\;}^{b}$	1.2	0 (<×2)
CLP	23.75 ± ${7.87\;}^{a}$	2.94	2 (≥×2)
CLP + 10 mg/kg Rosuvastatin	12.05 ± ${2.38\;}^{b}$	1.50	0 (<×2)
CLP + 20 mg/kg Rosuvastatin	12.93 ± $2.85^{\;b}$	1.60	0 (<×2)

Statistical significance is shown as mean ± standard deviation. The alveolar septal thickness ratio in the sham group was designated as 1. ^a^ *p* < 0.001; Compared with the sham group. ^b^ *p* < 0.001; Compared with the CLP group. One-Way ANOVA/Dunn test.

**Table 7:** Immunohistochemical positivity score results (median with 25% and 75% interquartile range).

Group	Caspase-3 Positivity Score	NF-kB/p65 Positivity Score	8-OHdG Positivity Score
Sham	0 (0–0)	0 (0–0)	0 (0–0)
Sham + 10 mg/kg Rosuvastatin	0 (0–0)	0 (0–0)	0 (0–1)
Sham + 20 mg/kg Rosuvastatin	0 (0–0)	0 (0–1)	0 (0–1)
CLP	3 ${\left(3–3\right)}^{\;a\;}$	3 ${\left(3–3\right)\;}^{a}$	3 ${\left(2–3\right)\;}^{a}$
CLP + 10 mg/kg Rosuvastatin	0.5 ${\left(0–1\right)\;}^{b}$	0 ${\left(0–1\right)\;}^{b}$	0 ${\left(0–1\right)}^{\;\;b}$
CLP + 20 mg/kg Rosuvastatin	0 ${\left(0–1\right)\;}^{b}$	1 ${\left(0–1\right)\;}^{b}$	0 ${\left(0–1\right)\;}^{b}$

^a^ *p* < 0.001; Compared with the sham group. ^b^ *p* < 0.001; Compared with the CLP group. One-Way ANOVA/Dunn test.

## Data Availability

The data that support the findings of this study are available from the corresponding author upon reasonable request.

## Abbreviations

The following abbreviations are used in this manuscript:

ALI	Acute lung injury
CLP	Cecal ligation and puncture
MDA	Malondialdehyde
GSH	Reduced glutathione
ARDS	Acute respiratory distress syndrome
NF-κB/p65	Nuclear factor kappa B/65
TNF-α	Tumor necrosis factor-alpha
IL-6	Interleukin-6
IL-1β	Interleukin-1 beta
IL-10	Interleukin-10
ROS	Reactive oxygen species
SOD	Superoxide dismutase
GR	Glutathione reductase
GSH-Px	Glutathione peroxidase
CAT	Catalase
RNS	Reactive nitrogen species
8-OHdG	8-hydroxy-2′-deoxyguanosine
HMG-CoA	Hydroxy-3-methylglutaryl-CoA
TBARS	Thiobarbituric acid-reactive substances
DTNB	5,5-dithiobis-(2-nitrobenzoic acid)
H&E	Hematoxylin–Eosin
LDS	Lung damage score
IHC	Immunohistochemical
